# Combined effect of physical exercise and hormone replacement therapy on cardiovascular and metabolic health in postmenopausal women: A systematic review and meta-analysis

**DOI:** 10.1590/1414-431X2023e12241

**Published:** 2023-05-15

**Authors:** J.C. Sánchez-Delgado, A.M. Jácome-Hortúa, O.M. Uribe-Sarmiento, S.V. Philbois, A.C. Pereira, K.P. Rodrigues, H.C.D. Souza

**Affiliations:** 1 Laboratório de Cardiologia, Fisiologia e Fisioterapia, Departamento de Ciências da Saúde, Faculdade de Medicina de Ribeirão Preto, Universidade de São Paulo, Ribeirão Preto, SP, Brasil; 2 Universidad de Santander, Facultad de Ciencias Médicas y de la Salud, Bucaramanga, Colombia

**Keywords:** Exercise training, Secondary prevention, Metabolic diseases, Review, Menopause, Estrogens

## Abstract

The cardioprotective effect of postmenopausal hormone replacement therapy (HRT) has been demonstrated in several studies. Similarly, physical exercise has yielded positive results. However, the effects of their combination remain inconclusive. This review describes the combined effects of physical exercise and hormone therapy on cardiovascular and metabolic health in postmenopausal women. We searched the Scopus, Web of Science, PubMed, and Embase databases and included randomized controlled trials published up to December 2021 on the combined effects of physical exercise and hormone therapy on cardiovascular and metabolic health in postmenopausal women. We identified 148 articles, of which only seven met the inclusion criteria (386 participants; 91 [23%] HRT + exercise; 104 [27%] HRT; 103 [27%] exercise; 88 [23%] placebo). The combined treatment further decreased systolic blood pressure (SBP) compared to the isolated effect of aerobic training (AT) (mean difference [MD]=-1.69; 95% confidence interval [CI]=-2.65 to -0.72, n=73). Nevertheless, it attenuated the decrease in diastolic blood pressure (DBP) (MD=0.78; 95%CI: 0.22-1.35, n=73), and the increase in peak oxygen consumption (VO_2 peak_) promoted by exercise (AT + HRT=2.8±1.4 *vs* AT + placebo=5.8±3.4, P=0.02). The combination of AT and oral HRT improved SBP. However, AT alone seemed to have a better effect on physical fitness and DBP in postmenopausal women.

## Introduction

Menopause is characterized by ovarian hormone decline, a condition accompanied by an increased risk of cardiovascular disease, which is even greater in women with premature menopause resulting from a surgical procedure (1). The main conditions associated with this period of life are arterial hypertension and the potential development of heart failure (2). In addition, menopause is associated with increased body weight, specifically adipose tissue, often associated with insulin resistance, dyslipidemia, and increased cardiovascular and metabolic risks (3).

Postmenopausal hormone replacement therapy (HRT) is used to control the symptoms and mitigate the cardiovascular risks associated with menopause (4). In some studies, including the Women's Health Initiative (WHI) study, HRT was significantly associated with an increase in breast cancer incidence and other serious side effects (5,6). However, later studies attenuated these findings, pointing out that the WHI study results were not generalizable to patients after physiological menopause. In addition, most participants had their last menstrual period more than a decade before and 50% had cardiovascular risk factors, so the data were insufficient to demonstrate clear harm to women's health (7-11).

Postmenopausal HRT has been shown to be safe when considering individual risk/benefit of a patient (age, previous diseases, risk factors, etc.), dose, and route of application (7). The use of postmenopausal HRT in risk-free women younger than 60 years and within 10 years of the onset of menopause has been shown to have a beneficial effect on the cardiovascular system, reducing the prevalence of coronary heart disease and all-cause mortality (8-11). However, postmenopausal HRT use has declined, leaving many symptomatic women without effective treatment (8).

Physical exercise is considered the cornerstone of non-pharmacological treatment for women during the climacteric period and after menopause, showing improvements in blood pressure (BP), nitric oxide bioavailability, lipid profile, cardiovascular function, body weight, and cardiorespiratory fitness (2,6). One of the main cardiovascular effects of exercise in women with ovarian hormone decline is a decrease in BP (12-17). Experimental studies in hypertensive ovariectomized rats have shown that BP reduction is associated with increased cardiac vagal tone and/or decreased sympathetic tone and improved baroreflex sensitivity (18-22).

The isolated beneficial effects of HRT on cardiometabolic health and adaptation to the women's condition and physical exercise at this stage of life have been widely recognized (23-25). Therefore, it is important to investigate the combined effect of these two interventions, considering that the physiological responses and adaptations induced by physical training could modulate the pharmacokinetics and pharmacodynamics of hormone treatment without excluding the possibility of bidirectional interactions (26,27).

Thus, data review and meta-analysis are important methods to investigate whether these interactions exist and how large their effects are. Moreover, HRT has several individual factors such as type, dose, and route of administration, similar to physical exercise (type, mode, duration, and intensity). These factors may help prioritize the most effective treatments or indicate the need for further research on different interventions to promote postmenopausal cardiovascular health (27).

To the best of our knowledge, there is no published systematic literature review showing the combined effects of physical exercise and postmenopausal HRT on cardiovascular and metabolic health, which is the main objective of this study. We hypothesized that the combination of HRT and physical exercise would lead to greater cardiometabolic health gains than the isolated treatments.

## Material and Methods

This systematic review was performed following the Preferred Reporting Items for Systematic Reviews and Meta-analyses (PRISMA) statement guidelines (http://www.prisma-statement.org) to identify randomized controlled trials (control or comparison arms), published up to December 2021, written in English, Portuguese, or Spanish, on the effects of aerobic physical training (AT) and postmenopausal HRT on cardiovascular and metabolic variables. Animal studies and studies involving women cohorts before and after menopause and women undergoing chemotherapy or radiation were excluded from the analysis.

The Scopus, Web of Science, PubMed, and Embase databases were searched using keywords extracted from Medical Subject Headings (MeSH) or EMTREE, with the Boolean descriptors “OR” within the word group and “AND” to combine terms related to population, intervention, control group, and outcomes. The research was developed considering parentheses, quotation marks, Boolean operators, and truncations used by each database. The complete search strategy is shown in Supplementary Table S1.

The selected keywords used for the database search were as follows: population (postmenopause, post menopause, postmenopausal female, postmenopausal period, postmenopausal women); type of study (randomized clinical trial, controlled clinical trial, Clinical Trial, Comparative Study, Clinical Trials as Topic, random*, controll*, intervention study, experimental study, trial, trials, evaluat*, repeat*, compar*, and controlled clinical comparison); intervention (exercise, exercise therapy, exercise training, and physical exercise); control or comparison group (anti-hypertensive agents, anti-hypertensive drug, estrogen replacement therapy, estrogen administration, and estrogen treatment); and results (autonomic nervous system, heart rate, blood pressure, blood tension, arterial baroreflex, tilt-table test, heart rate variability, blood pressure variability, cardiac function, heart function, heart muscle function, myocardial function, cardiac remodeling, heart ventricle remodeling, ventricular remodeling, heart disease, heart dysfunction, weight, metabolic diseases, nutritional and metabolic diseases, lipids, arterial stiffness, atherosclerosis, arteriosclerosis, cardiovascular diseases, hypertension, diabetes mellitus, obesity, and coronary artery disease).

Three researchers selected the abstracts of potentially eligible articles and performed a full-text review to confirm compliance with the eligibility criteria. Analysis of the articles was performed independently by the evaluators and, in case of discrepancies, a fourth evaluator made the decision.

Subsequently, the methodological quality of each selected article was determined using the PEDro scale (www.pedro.org.au) (28,29). This scale comprises 11 items that assess eligibility, randomization, blinding, allocation masking, group comparability at baseline, masking, intent-to-treat analysis, and adequate follow-up outcomes. The total PEDro score is the sum of each criterion met and ranges from 1 to 11. A higher score indicates a better methodological quality of the study. A total score of 10-11 points indicates excellent, 7-9 points good, 5-6 points fair, and <5 points poor methodological quality (30). The quality or certainty of the evidence was rated with the GRADEPro tool regarding the risk of bias, imprecision, inconsistency, indirectness, and publication bias (31,32).

Finally, information related to sample size, age, type of intervention, and main results in the selected articles were extracted and analyzed. The synthesis and analysis of this information were narrative and qualitative, respectively. When possible, meta-analyses were performed using RevMan 5.4 to compare the mean differences and 95%CI for continuous outcomes between the intervention and control/comparison trial groups. We performed the following subgroup analysis based on the results: 1) combined treatment (HRT + exercise) versus physical exercise alone, and 2) HRT + exercise versus HRT alone. The protocol for this systematic review was registered with PROSPERO database (CRD42021102909).

## Results

**Figure 1:**
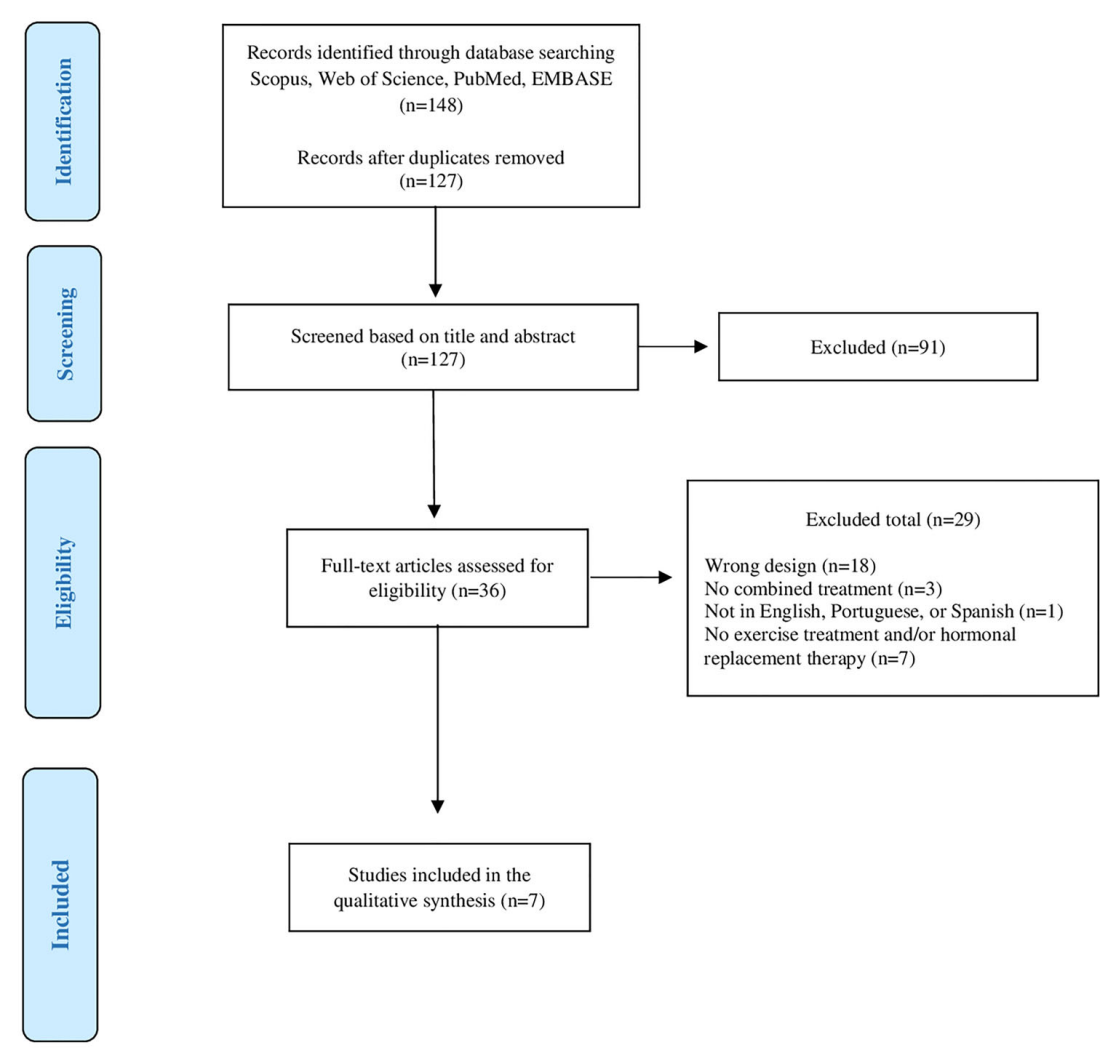
Flow diagram of study selection process.

A total of 148 studies were identified, five of which (seven publications) met the eligibility criteria and were analyzed (Figure 1). All the studies used randomization, baseline comparison, inter-group comparison, measures of variability, and assessment of at least one key outcome in more than 85% of the subjects initially allocated, conforming to the PEDro scale. Blind distribution and intention-to-treat analyses were not used in any of the studies (Supplementary Table S2). As to the quality of the evidence at the level of the meta-analysis outcomes, Supplementary Table S3 shows very low or low quality.

The analyzed studies included 23-101 physically inactive postmenopausal women without a history of cardiovascular diseases aged 48-58 years (33-39). One study evaluated the combination of resistance training and AT (33) and the others investigated the effects of AT (34-39). The HRT used was orally administered and included estradiol valerate (36-39), unopposed estrogen, and/or progestins (33-35).

### Outcomes

The analyzed studies reported effects on functional capacity, body composition, BP, blood flow, sympathetic activity, lipoprotein levels, and muscle strength. Supplementary Table S4 provides a complete list of the evaluated results.

#### Combined effect of aerobic physical exercise and postmenopausal HRT on functional capacity

**Figure 2:**
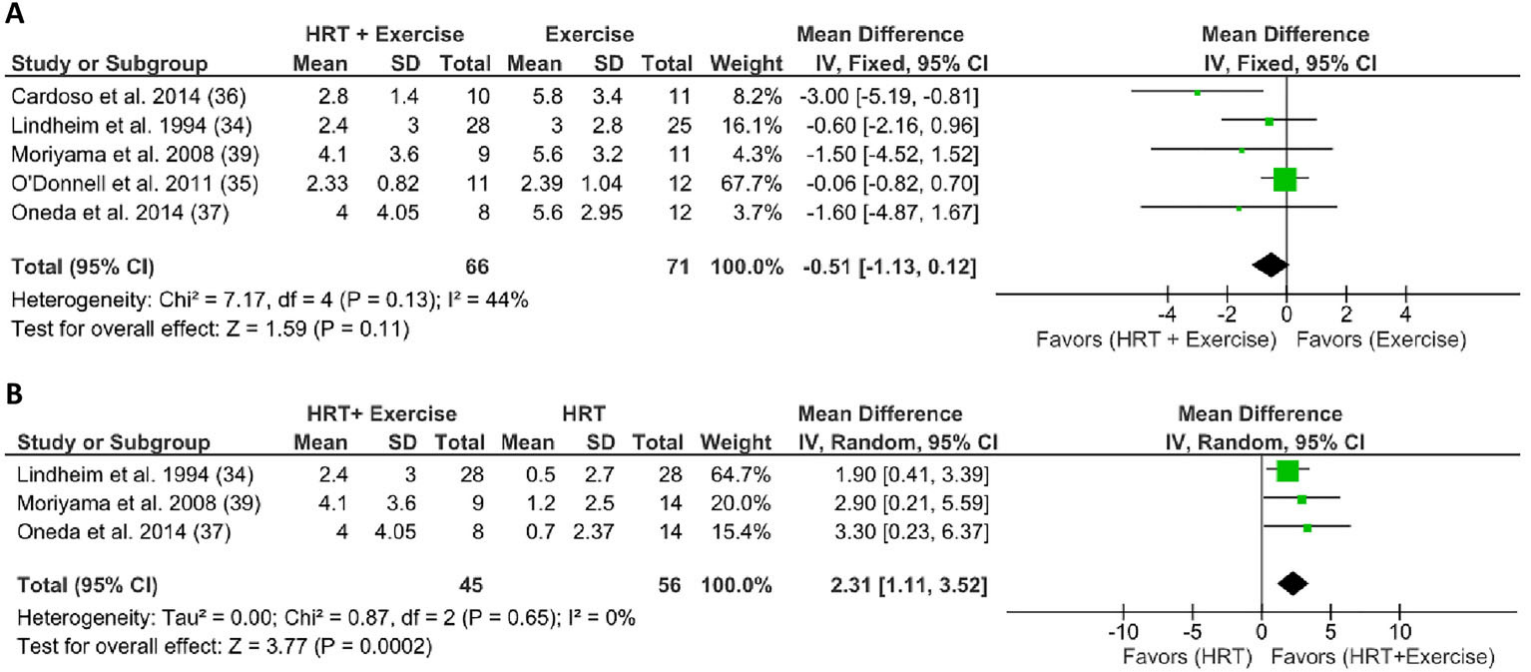
Forest plot of meta-analysis results presented as pooled standard mean differences with 95%CI for changes in oxygen uptake at peak exercise of experimental (subjects with HRT and exercise) and control [subjects with exercise only (**A**) or HRT only (**B**)] groups. The combined effects are plotted with black diamonds. IV: inverse variance; HRT: hormonal replacement therapy.

Five studies (71%) reported results related to physical fitness. These included AT performed 3 times a week with moderate intensity and oral HRT with 1 mg/day estrogen (36,37,39) or 0.625 mg/day equine conjugated estrogen (34) for 26 weeks. Only one trial included five weekly training sessions, with 1 mg/day oral estrogen for 12 weeks (35). Cardoso et al. (36) showed that HRT attenuated the increase in oxygen consumption (VO_2peak_) promoted by AT (AT + HRT=2.8±1.4 *vs* AT + placebo=5.8±3.4, P=0.02). The VO_2peak_ meta-analysis did not show any change that favored the combined treatment (HRT + exercise) over exercise alone. However, the combined treatment had better results than HRT alone (MD=2.31; 95%CI: 1.11 to 3.52; P=0.002; heterogeneity I^2^: 0%) (Figure 2).

#### Combined effect of physical exercise and postmenopausal HRT on blood pressure

**Figure 3:**
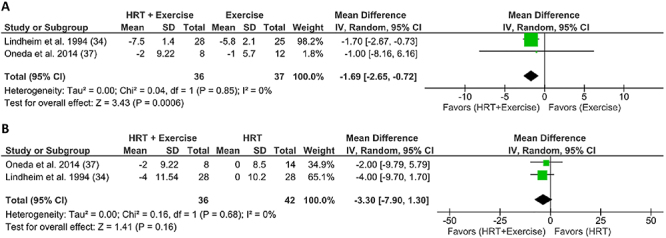
Forest plot of meta-analysis results presented as pooled standard mean differences with 95%CI for changes in systolic blood pressure of experimental (subjects with HRT and exercise) and control [subjects with exercise only (**A**) or HRT only (**B**)] groups. The combined effects are plotted with black diamonds. IV: inverse variance; HRT: hormonal replacement therapy.

**Figure 4:**
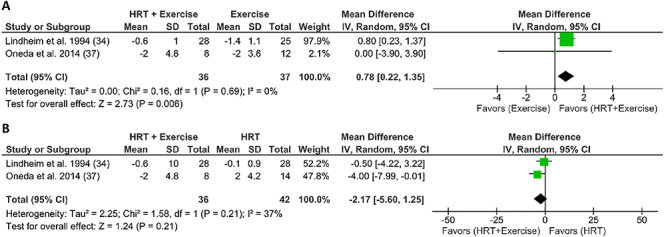
Forest plot of meta-analysis results presented as pooled standard mean differences with 95%CI for changes in diastolic blood pressure of experimental (subjects with HRT and exercise) and control [subjects with exercise only (**A**) or HRT only (**B**)] groups. The combined effects are plotted with black diamonds. IV: inverse variance; HRT: hormonal replacement therapy.

Two studies (29%) reporting BP results included AT at three sessions per week, as well as 1 mg/day oral estrogen (37) or conjugated equine estrogen at 0.625 mg/day (34) for 26 weeks. Figure 3A shows that the combined treatment further decreased SBP compared with AT alone (MD=-1.69; 95%CI: -2.65 to -0.72; P=0.0006; I_2_: 0%, n=73). In contrast, Figure 4A shows that the combined treatment increased DBP levels compared to exercise alone (MD=0.78; 95%CI: 0.22 to 1.35; P=0.006; I_2_: 0%, n=73).

## Discussion

The current study showed that compared with exercise alone, combined treatment with AT and oral HRT may have a better effect on SBP. However, AT alone seems to have better effects on DBP and physical fitness than combined treatment in healthy postmenopausal women.

Most postmenopausal women have decreased exercise tolerance, a condition associated with increased mortality. This may be influenced by increased adiposity, reduced basal metabolic rate, and lower peripheral arterial flow induced by reduced estrogen and nitric oxide levels, as well as reduced skeletal muscle mass and strength (40-42). However, the results regarding the association between HRT and physical fitness are controversial; most studies have shown that this combination does not influence VO_2max_ (43-45). Some authors, on the other hand, have shown that HRT increases arterial compliance during exercise, possibly facilitating the attainment of favorable oxygen consumption (46). Others have pointed out that ovarian hormone deprivation in the first stages of menopause does not seem to significantly influence functional capacity (47). In contrast, Cardoso et al. (36) suggested that the attenuation of gains in VO_2peak_ as a result of HRT may be justified by the increase in mitochondrial oxidative stress generated by HRT and its modulation of sympathetic tone, leading to lower secretion of catecholamines and limiting the production and use of glucose, mainly during the high intensities reached in the effort tests (48-51).

Results in the literature diverge on the effects of the combined treatment on exercise tolerance (44,52-54). Our meta-analysis showed that aerobic exercise associated with HRT presented better results than sedentary behavior and HRT (Figure 2B), largely justified by the adaptations promoted by aerobic physical training on the systems that transport and absorb oxygen. Specifically, exercise increased left ventricular compliance and force of contraction, cardiac output, red blood cell levels, capillary and mitochondrial density, and the synthesis of vasodilating substances such as nitric oxide and prostacyclin (51,54-58).

The positive effect of the combined treatment on SBP at rest (Figure 3A) can be explained, at least in part, by the cardioprotective effects of exercise, such as decrease in the activity of the renin-angiotensin aldosterone system (RAAS) and sympathetic nervous system, and increase in endothelial dilating factors. These effects may be reinforced and/or complemented by HRT, potentiating mitochondrial function (49) and favoring endothelial homeostasis and nitric oxide-mediated vasodilation (59,60).

However, there is evidence linking the increase in BP to the hepatic first-pass clearance of estrogen (61,62), possibly explaining the subtle increase in DBP in the groups that received the combined treatment of AT + HRT (Figure 4A). Specifically, oral estrogen administration, particularly conjugated equine estrogens, has been reported to have a stimulatory effect on the RAAS, increasing liver renin substrates and favoring vasoconstriction (61-63), a condition that mainly determines DBP and that does not seem to be affected by AT.

Since the articles analyzed in this study included aerobic exercise, it would be pertinent to investigate the effects of alternative training models that seem to have positive effects on functional physical capacity (64-67), BP control (68-72), and other cardiometabolic parameters of clinical relevance in postmenopausal women. Similarly, HRT must be considered because its effects can change according to the type of hormone, dose, and route of administration. For example, transdermal estrogens have shown better effects on BP compared with oral HRT (73). Additionally, randomized trials should be carried out to evaluate other cardiometabolic variables sensitive to ovarian hormone deprivation that are related to an increased risk of cardiovascular disease and mortality in postmenopausal women, such as those related to cardiovascular autonomic modulation, inflammatory markers, and arterial stiffness (16-19,74).

The limitations of our study must be considered. First, some reports reviewed in the current study did not define the type of menopause assessed (33,34), whereas others had a mix of surgical and physiological menopause populations (35-38). Therefore, our findings cannot distinguish between surgical or physiological post-menopause, and our results may have been affected by a meta-analysis bias. Because of the low number of analyzed randomized clinical trials, the low quality of the evidence, and the low number of participants in most of the articles, the results of our review need to be considered with some caution.

### Conclusion

Aerobic physical training associated with oral HRT may have a better effect on SBP compared with exercise alone; however, for DBP and physical fitness, physical exercise alone seems to be better for healthy postmenopausal women. Further clinical trials are needed, including larger samples and greater methodological rigor, alternative physical exercise treatments, other HRT administration modalities, and longer intervention periods. Changes in lifestyle, particularly physical activity levels and nutritional quality, continue to be the pillars for maintaining cardiometabolic health in this population.

## Supplementary Material

Click to view [pdf].
